# *Sumakuru*, a deeply-diverging new genus of lyssomanine jumping spiders from Ecuador (Araneae: Salticidae)

**DOI:** 10.3897/zookeys.614.9368

**Published:** 2016-09-01

**Authors:** Wayne P. Maddison

**Affiliations:** 1 Departments of Zoology and Botany and Beaty Biodiversity Museum, University of British Columbia, 6270 University Boulevard, Vancouver, British Columbia, V6T 1Z4, Canada

**Keywords:** Jumping spider, Lyssomaninae, molecular phylogeny, new genus, new species

## Abstract

The lyssomanine jumping spider genus *Sumakuru* **gen. n.** is here described for *Sumakuru bigal* **sp. n.**, from the Bigal River Biological Reserve in Ecuador. Known from a single male, the embolus of the palp takes the form of a smoothly arching curve, and appears fully mobile, being connected to the tegulum by a thin sclerite and a twisted hematodocha. Data from four gene regions (28S, 16SND1, CO1, *wingless*) indicate that *Sumakuru* is the sister group to all other sampled lyssomanines, diverging deeply on the stem lineage of the clade of other known lyssomanines. Unlike previous molecular results, the sampled species of *Lyssomanes* Hentz, 1845 are supported as monophyletic, with *Chinoscopus* Simon, 1900 as the sister to *Lyssomanes*.

## Introduction

The distinctive lyssomanine jumping spiders include two described genera, *Lyssomanes* Hentz, 1845 and *Chinoscopus* Simon, 1900, both neotropical (Galiano 1962, 1980; Logunov and Marusik 2003; Maddison 2015). Recent molecular phylogenetic analyses show, as expected, that they form an isolated group (Maddison et al. 2014), related to the Asemoneinae or Spartaeinae (Su et al. 2007; Maddison et al. 2014). However, these analyses suggest that the living lyssomanine species represent a recent radiation, with relatively short branches separating the sample of 10 diverse species of *Lyssomanes* and one of *Chinoscopus* (Maddison et al. 2014: figs 15–17), which together sit atop a long ancestral branch connecting them to other salticids. This presents a challenge: are there any living lyssomanines yet to be discovered that diverge deeply, breaking up this long branch as *Thrandina* Maddison, 2006 does for the lapsiines, for instance?

A single male of a new species of lyssomanine from the Bigal River Biological Reserve in Ecuador bears a distinctive palp, but it appears to be within the range of morphological diversity in *Lyssomanes* (Galiano 1980, Logunov 2014). Molecular data, however, show it to be remarkably distinct from the other species sampled to date, lying well outside the clade of *Lyssomanes* plus *Chinoscopus*. It is therefore described here as the new genus *Sumakuru*.

## Material and methods

The preserved specimen was examined under both dissecting microscopes and a compound microscope with reflected light. Drawings were made with a drawing tube on a Nikon ME600L compound microscope.

Terminology is standard for Araneae. All measurements are given in millimeters. Descriptions of color pattern are based on the alcohol-preserved specimen. Carapace length was measured from the base of the anterior median eyes not including the lenses to the rear margin of the carapace medially; abdomen length to the end of the anal tubercle. The following abbreviations are used: ALE, anterior lateral eyes; PLE, posterior lateral eyes; PME, posterior median eyes (the “small eyes”).

DNA sequences of the genes or gene regions 28S, *wingless*, CO1 and 16SND1 were obtained from the holotype of *Sumakuru bigal* using the protocols of Zhang and Maddison (2013) and Maddison et al. (2014). The first two of these genes are nuclear, the last two mitochondrial. These sequences were added to data from 78 taxa borrowed from the non-salticoid dataset of Maddison et al. (2014), including 10 *Lyssomanes*, 1 *Chinoscopus*, 7 non-salticids, 2 Onomastinae, 5 Asemoneinae, 32 Spartaeinae, 6 Hisponinae, and 15 Salticinae. The Genbank accession numbers of the borrowed data can be obtained from the table Supplementary Material S1 of Maddison et al. (2014), following the voucher identification codes that are here appended to the taxon names in the figures of the phylogeny.

Prior to phylogenetic analysis, multiple sequence alignment was done for 28S and the noncoding portion of 16SND1 with MAFFT (Katoh et al. 2002, 2005) using the LINSI option (--localpair --maxiterate 1000), run via Mesquite (Maddison and Maddison 2015b). Mesquite was used to color the matrix via the option ‘‘Highlight Apparently Slightly Misaligned Regions’’ so as to identify regions that needed correction. Alignment of coding regions was easily done by eye through translation to amino acids.

Maximum likelihood phylogenetic analyses were run using RAxML version 8.2.8 (Stamatakis 2014), run via Mesquite’s Zephyr package (Maddison and Maddison 2015a). 100 search replicates were performed to find the maximum likelihood tree, while 1000 bootstrap replicates were done to assess repeatability of the results (1 search replicate per bootstrap replicate). The analyses partitioned the data (see below) and used GTR+G+I for each of the partitions. Analyses were done on the complete dataset of four genes, as well as on two partial datasets, to determine if their results were concordant: the first partial dataset had 28S only, while the second had the other three gene regions (16SND1, CO1, *wingless*).

To choose a partitioning scheme for the RAxML analyses, PartitionFinder 1.1.1 (Lanfear et al. 2012) was given an all-genes matrix with 11 partitions (28S, non-coding region of 16SND1, and first/second/third codon positions for each of ND1, CO1, and *wingless*). Parameters were branchlengths = linked; models = raxml; model_selection = BIC; search = greedy.

Alignments and trees are deposited in the Dryad data repository (http://dx.doi.org/10.5061/dryad.2g8j2).

## Taxonomy

### Sumakuru

Taxon classificationAnimaliaAraneaeSalticidae

Maddison gen. n.

http://zoobank.org/AF495AC9-F56E-44B3-AB88-D1D73AA1089D

#### Type species.

*Sumakuru bigal* Maddison, sp. n.

#### Etymology.

From the Quechua *sumak*, “great, marvellous” and *uru*, “spider”. The same root *sumak* is the source of the name of the volcano Sumaco, from whose southeastern slopes the type species is known. *Sumakuru* is to be treated as grammatically masculine.

#### Diagnosis.

Delicate, pale, and long legged as in other lyssomanines, but with a distinctive palp in which the smoothly arching embolus is connected to the tegulum by a thin sclerite and twisted hematodocha (Fig. 3 arrow). The carapace is narrow, but higher than in *Chinoscopus*; the male chelicerae are simple and relatively short, not long and diverging as in many species of *Lyssomanes*. While the form of the embolus is unique among known lyssomanines, the other features cited are not fully distinctive, as some *Lyssomanes* have a narrow carapace and short male chelicerae. It is unfortunate that we do not yet have morphological synapomorphies to distinguish each of *Sumakuru*, *Lyssomanes* and *Chinoscopus* from one another. However, the molecular data strongly support the distinction of *Sumakuru* from other lyssomanines, as discussed below. Some specific sites in the alignments submitted to Dryad at which *Sumakuru* is unique are: at site 562 of the 28S alignment, *Sumakuru* has T instead of G or A; site 861 of 28S, A instead of G; site 589 of 16SND1, A instead of T (thus rendering the 10th amino acid of ND1 translated as asparagine instead of either isoleucine or valine). Comments on monophyly are given under “Phylogeny and Discussion”.

**Figures 1–11.**
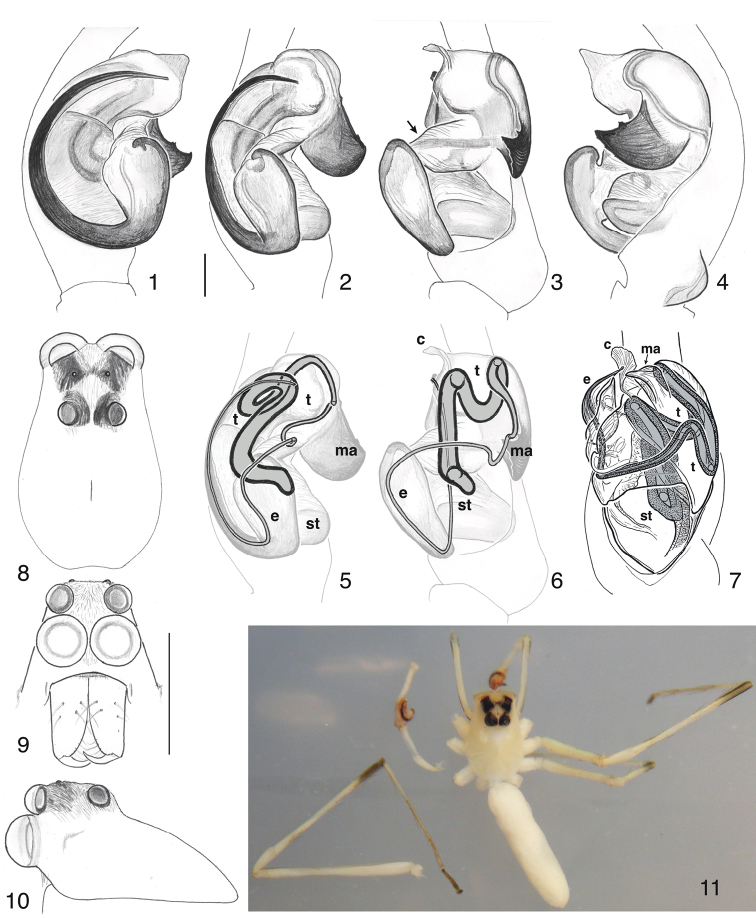
*Sumakuru bigal* sp. n., holotype, except **7** (*Lyssomanes* for comparison). **1–6** Left palp. **1** Prolateral view **2** Oblique prolateral-ventral view (scale bar 0.1 mm) **3** Ventral view **4** Retrolateral view **5** Oblique prolateral-ventral view showing path of spermophore (as seen by clove oil clearing) and interpretation of parts **6** Same, ventral view **7** Trypsin-cleared left palp of *Lyssomanes viridis*, ventral view, showing path of spermophore **8** Carapace, dorsal view **9** face (scale bar 1.0 mm) **10** Carapace, lateral view **11** Photograph of holotype, with left palp and left second leg separated. Abbreviations: **e** = embolus; **c** = conductor; **ma** = median apophysis; **t** = tegulum; **st** = subtegulum.

### Sumakuru bigal

Taxon classificationAnimaliaAraneaeSalticidae

Maddison sp. n.

http://zoobank.org/29AE8C1D-8C6F-4140-A4D6-1612B0CC1C77

Figs 1–11

#### Type material.

Holotype: male, ECUADOR: Orellana: Río Bigal Reserve, Mirador Trail. S 0.5282 W 77.4195. 950 m elev. 2–4 November 2010. W & D Maddison, M Vega, M Reyes. WPM#10-043. DNA voucher d448. The specimen pertains to the Museum of Zoology, Pontificia Universidad Católica, Quito, Ecuador (QCAZ), but is currently held in the Spencer Entomological Collection at the Beaty Biodiversity Museum, University of British Columbia
(UBC-SEM).

#### Etymology.

Based on the type locality.

#### Diagnosis.

The distinct arching spiral of the embolus (Fig. 1) is unlike any other known lyssomanine, except perhaps *Lyssomanes tarmae* Galiano, 1980, whose embolus is thicker. The carapace is relatively narrow, and the dark markings on the tarsi and ends of the tibiae of legs 2 and 3 are distinctive (Fig. 11).

#### Notes.

The single male was found by beating understory vegetation in a relatively open tropical rainforest along a ridge. It landed injured on the beating sheet, having lost most of its legs. The preserved specimen now has both palpi, but just 3 legs: the second legs on both sides, and the third leg on the right side.

#### Description.

*Male* (holotype, DNA voucher d448). Carapace length 1.7; abdomen length 2.8. Chelicera (Fig. 9): modest in size, vertical. Teeth not examined for fear of breaking this singular specimen, but no large or prominent teeth projecting beyond the endites. Palp (Figs 1–6): segments long, such that femur is as long as the carapace. Tip of cymbium elongate, extending well distal to bulb (Fig. 11). The bulb’s basic configuration is much like that of *Lyssomanes viridis* (Walckenaer, 1837) (Fig. 7). The subtegulum is exposed at the proximal side of the bulb, the tegulum occupies the distal retrolateral part of the bulb, and the embolus is on the prolateral side (Figs 5–6). The embolus is connected to the tegulum by a narrow sclerite and twisted hematodocha (arrow in Fig. 3). A diaphanous conductor is terminal (c in Figs 5–6). A broad blade-shaped apophysis, its apparent homolog interpreted as the median apophysis by Galiano (1962 figs 1–2), arises from the retrolateral side of the tegulum (ma in Figs 5–6). The spermophore begins proximally, in the subtegulum, then moves distally into the tegulum (Figs 5–6) then has a loop extending proximally into the tegulum, before coming into the retrolateral side of the tegulum. From there the duct runs through a narrow bridge of sclerite and hematodocha to cross over the face of the bulb to the prolateral embolus (Figs 5–6). The same configuration of the spermophore is seen in *Lyssomanes* (Fig. 7). Legs long, typical for lyssomanines. Carapace (Figs 8–10): narrow, with fovea displaced to the posterior. ALE directly above AME (Fig. 9). Color in alcohol (Fig. 11): pale, almost white, except for black eyes and appendages as noted (Fig. 11). Black pigment on the tarsus and distalmost quarter of the tibia of legs 2 and 3. On the palp is some black pigment on the retrolateral edge of the trochanter and femur, and the distal prolateral edge of the femur. Some pale scales clothe the dark ocular region (Figs 8–10).

## Phylogeny and Discussion

Sequences of the holotype of *Sumakuru bigal* were obtained for 28S (GenBank accession number KX578224), 16SND1 (KX578225), CO1 (KX578226) and *wingless* (KX578227). Alignment by MAFFT appeared reasonable except for a few obvious shifts of 3 to 7 nucleotides near the starts or ends of some outgroup sequences, which were corrected by hand (28S Aelurillus cf. ater, *Cucudeta zabkai*, *Onomastus nigrimaculatus*, *Cocalodes longicornis*; 16SND1 *Afromarengo* sp., *Naphrys pulex*).

PartitionFinder determined the best partitioning scheme was to keep all partitions separate except to group the noncoding portion of 16SND1 with the first codon position of ND1, and to group first and second codon positions of *wingless*. For all of these GTR+I+G was determined as the best model, except for ND1 second position (GTR+G).

**Figures 12–14.**
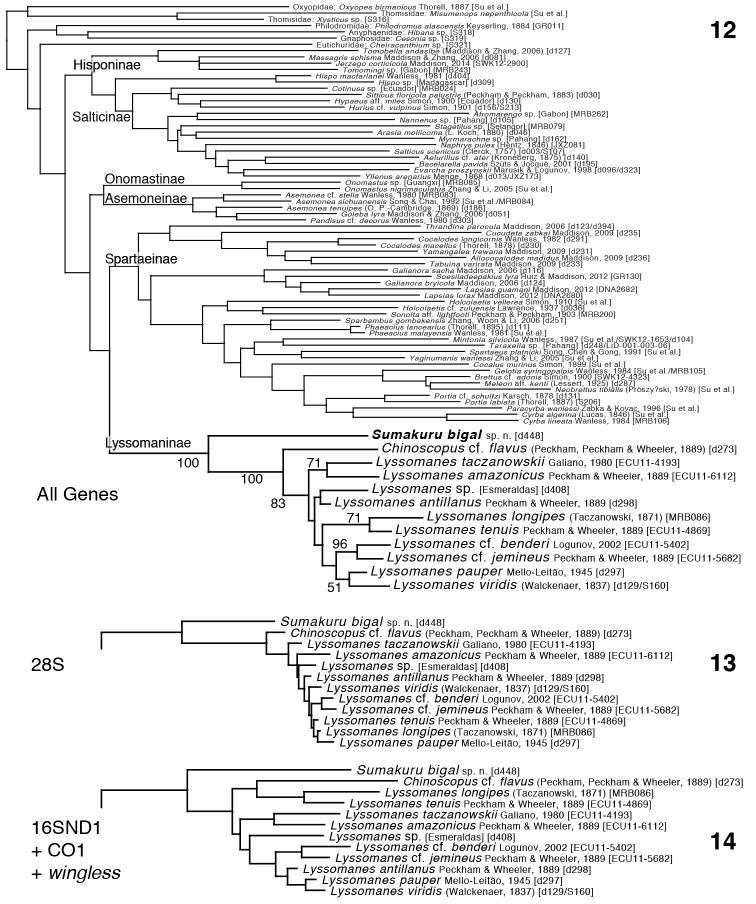
Maximum likelihood phylogenetic trees from RAxML analyses. Appended to taxon names are the identification codes of voucher specimens used **12** Phylogeny from all 4 genes concatenated; bootstrap percentages shown for lyssomanines only **13** Lyssomanine portion of phylogeny from 28S alone **14** Lyssomanine portion of phylogeny from three gene regions concatenated, 16SND1+CO1+*wingless*.

Phylogenetic trees inferred are shown in Figs 12–14. Among the outgroups (i.e. all taxa other than lyssomanines), the All Genes tree in most respects matches that of Maddison et al. (2014), though there are a few differences, including the non-monophyly of hisponines, perhaps because the sample here of non-lyssomanine taxa and genes is reduced compared to theirs.

*Sumakuru* is placed with strong support as a lyssomanine and as the sister group to *Chinoscopus* + *Lyssomanes*. The monophyly of the Lyssomaninae is supported in 100% of the bootstrap replicates in the All Genes analysis (Fig. 1), and in the separate 28S and 16SND1+CO1+*wingless* analyses (Figs 13, 14). Likewise, the monophyly of *Lyssomanes* + *Chinoscopus* (excluding *Sumakuru*) is supported in the All Genes analysis (100%), 28S analysis, and 16SND1+CO1+*wingless* analysis. Unlike the analyses of Maddison et al. (2014), which embedded *Chinoscopus* within *Lyssomanes*, our All Genes analysis gives reasonable support to the monophyly of *Lyssomanes* (83%).

The molecular phylogeny provides sufficient reason to recognize *Sumakuru* as a distinct genus, given the study’s inclusion of the type species of *Lyssomanes* (*Lyssomanes viridis*) and the close similarity between the species of *Chinoscopus* studied and the type species *Chinoscopus gracilis* (Taczanowski, 1872). However, the molecular phylogeny does not provide much support for the full monophyly of *Lyssomanes* as currently composed, as we lack molecular data for many species now placed in *Lyssomanes*, and there has been little phylogenetic work using morphology. The delicate body and unusual genitalia of *Chinoscopus* can be interpreted as synapomorphies for that genus, but no such synapomorphies are known for *Lyssomanes* as a whole. Although many excellent figures of male palps of *Lyssomanes* species have been published (e.g. Galiano 1980, Logunov and Marusik 2003, Logunov 2014), the palpi are complex and diverse, and few species have received detailed morphological interpretations. There has therefore been little proposed about phylogenetic relationships within *Lyssomanes*, except for Galiano’s (1980) species groups.

That *Lyssomanes* could be monophyletic receives some support from the fact that the ten *Lyssomanes* species in the molecular phylogenetic analysis cover a broad spectrum of the genus, including representatives of Galiano’s (1980) *longipes*, *robustus*, *antillanus*, *amazonicus*, *viridis* and *jemineus* species groups. However, the genus includes 90 described species showing great diversity in genitalic morphology (Galiano 1962, 1980, 1984, 1996; Brignoli 1984; Jiménez and Tejas 1993; Logunov 2000, 2002, 2014, 2015; Logunov and Marusik 2003). Nine of Galiano’s 15 species groups are not yet represented in a phylogenetic analysis. Among these other species groups yet to be studied may be other species that belong in *Sumakuru*, or as separate deeply diverging lineages.

Among the *Lyssomanes* species not studied in the molecular phylogeny are some whose similarities to *Sumakuru bigal* are likely convergent. For instance, *Lyssomanes spiralis* F.O. Pickard-Cambridge, 1900 also has a spiraled embolus (Galiano 1980: figs 138–199). However, the body is otherwise unlike *Sumakuru*, robust and with long jaws (F.O. Pickard-Cambridge 1900). Its apparent relative Lyssomanes cf. jemineus groups with *Lyssomanes* in the molecular phylogeny (*Lyssomanes jemineus* and *Lyssomanes spiralis* were placed by Galiano 1980 in the same species group).

Some species currently placed in *Lyssomanes* stand out as unusual, therefore possibly falling outside of *Lyssomanes*, either as relatives of *Sumakuru bigal*, or as separate deeply-branching lineages. *Lyssomanes tarmae* Galiano, 1980 has a distinctive palp with an arching embolus that resembles that of *Sumakuru bigal*, though more robust. *Lyssomanes elongatus* Galiano, 1980, known only from the female, has a carapace shape similar to *Sumakuru bigal*. Although not obviously similar to *Sumakuru bigal*, the species *Lyssomanes romani* Logunov, 2000 may have a special phylogenetic position, based on the median apophysis which is apparently articulated (Logunov 2000).

*Sumakuru* appears to have diverged relatively long ago from the lineage leading to *Lyssomanes* and *Chinoscopus*, given the length of the branch below the clade of *Lyssomanes* + *Chinoscopus* (Fig. 12) and the strong bootstrap support for that clade. The lapsiines of South America and the cocalodines of Australasia, both isolated non-salticine groups concentrated in moist lowland tropical rainforests, similarly have a deeply diverging lineage that has few known species and that exists in higher elevation tropics. In the lapsiines, the phylogenetically isolated lineage is the unusual *Thrandina* with its large PME. In the cocalodines, it is the almost ant-like ground-dwelling *Cucudeta* Maddison, 2009 (though the position of *Depreissia* Lessert, 1942 within the cocalodines is unclear, Maddison et al. 2016). In the lyssomanines, the newly-discovered deeply diverging lineage is *Sumakuru*. These three unusual lineages all live close to the equator (among them, the maximum known latitude is 6°) and at mid to high elevations (all are from 1000 to 2500 m elevation). To find distinctive undiscovered lineages of salticids, it may be especially important to search at 1000–2500 m elevation in equatorial regions.

## Supplementary Material

XML Treatment for Sumakuru

XML Treatment for Sumakuru bigal
